# Is the performance of acute appendectomy at different times of day equal, in terms of postoperative complications, readmission, death, and length of hospital stay? A Swedish retrospective cohort study of 4950 patients

**DOI:** 10.1007/s00068-023-02395-6

**Published:** 2023-12-04

**Authors:** Petter Nyström, Martin Nordberg, Lennart Boström

**Affiliations:** 1 Department of Surgery, South General Hospital (Södersjukhuset), Stockholm, Sweden; 2 Department of Clinical Science and Education, Karolinska Institutet, Stockholm, Sweden

**Keywords:** Appendicitis, Appendectomy, After-hours care, Postoperative complications, Length of stay

## Abstract

**Purpose:**

Appendicitis is one of the most common acute surgical conditions globally, and hence appendectomy is a common procedure performed around the clock in many hospitals. The aim of the current study was to determine whether acute appendectomy due to acute appendicitis performed during day, evening, and night was equally safe, in terms of postoperative complications, readmission, death, and length of hospital stay.

**Methods:**

A retrospective single-center cohort study, using a local quality register of all consecutive acute appendectomies performed at the Department of Surgery, Södersjukhuset, Stockholm, Sweden. During the study period from December 2015 to August 2022, 4950 patients were included. Risk of complications, readmission, and death were determined using multivariable logistic regression models. Association with length of hospital stay was determined using multiple linear regression.

**Results:**

There was no significant difference in the associated risk of postoperative complications, readmission within 30 days, or death, regardless of when appendectomy was performed. Using daytime surgery as reference, hospital stay was shortened by 4.21 h (*P* = 0.008) for evening surgery and by 6.71 h (*P* < 0.001) for nightly surgery.

**Conclusion:**

Risks of postoperative complications, readmission, and death were similar regardless of when acute appendectomy was performed. However, surgery during evening and night was associated with shortened hospital stay, as compared to daytime surgery.

## Introduction

Acute appendicitis is a common cause of acute abdominal pain, with an estimated lifetime risk of 6.7% in women and 8.6% in men [1]. Surgical removal of the appendix, appendectomy, was introduced as treatment of acute appendicitis in 1894 [2] and it was initially thought that unless an inflamed appendix was removed surgically, it would sooner or later perforate [3]. However, current evidence states that cases of uncomplicated appendicitis can be treated with antibiotics alone or even resolve without treatment, although risk of complications is higher with an initial non-surgical approach. Also, a quarter of the patients who initially are treated conservatively will still need to undergo appendectomy within a year [4]. Since acute appendicitis can be treated non-surgically in some cases, it is evident that a hasty appendectomy is not mandatory to prevent perforation and, thus, complications. Hence, the question of how urgently to perform appendectomy in patients with acute appendicitis has arisen [5]. A recent meta-analysis of roughly 150,000 patients by van Dijk et al. [6] has suggested that presumed uncomplicated acute appendicitis can be delayed up to 24 h from admission, without increased risk of perforation or postoperative complications. However, delaying appendectomy means delaying the patient´s relief of symptoms, thus probably increasing the patient´s use of opioids as well as prolonging hospital stay, with an increased cost as a consequence. Therefore, and to decrease patient discomfort, one could suggest that acute appendectomy should not be postponed more than necessary. It is therefore a clinically relevant question whether it is safe to operate patients with acute appendicitis after office hours, or if appendectomy should be postponed until the next day, which should be a surgically safe option [6, 7].

Although sleep deprivation is associated with a general negative impact on cognition [8] and night-time emergency surgery has been reported to be associated with increased postoperative morbidity [9] and mortality [10] rates, studies show that technical performance is preserved in sleep-deprived surgeons [11, 12]. Recent studies suggest that night-time appendectomy is safe and not associated with increased risks as opposed to daytime surgery [13, 14].

The aim of the current study was to further examine acute appendectomy performed at different times around the day and the association with postoperative outcome, including complications, readmission, death, and length of hospital stay.

## Materials and methods

### Study hospital

The current study was designed as a retrospective cohort study and was carried out between December 2015 and August 2022, at Södersjukhuset (Stockholm South General Hospital), Stockholm, Sweden. It is one of the seven emergency hospitals in Stockholm County and has a referral area of roughly 700,000 adult inhabitants. In 2021, the total number of patients presenting in the emergency department was 112,826 (of which 21,600 were children aged < 18 years) and 49,781 patients were admitted to the hospital. As of November 2022, the Department of Surgery had 84 inpatient beds and 345 employees, of which 66 were doctors.

Previous to September 2016, only acute appendicitis patients aged > 14 years were treated at the Department of Surgery at Stockholm South General Hospital. Younger Stockholm patients with surgical conditions were treated at Karolinska Universitestsjukhuset (Karolinska University Hospital), by pediatric surgeons. However, since September 2016, all pediatric acute appendicitis patients in the range of 10–14 years are treated Södersjukhuset. These patients are staying in a pediatric ward, but medical decisions and daily rounds are done by general surgeons.

At the Department of Surgery, office hours are between 7:30 and 16:00 Monday–Friday, non-Holidays. Night shifts are staffed by a doctor who is off during the day before as well as the day after the night shift. The night shift starts at 19:00 Mondays–Thursdays, at 16:00 on Fridays, and at 18:00 during weekends. Night shifts end at 07:30 during the week and at 8:00 on weekends. A night shift week is made up by three shifts, every other night, starting either on Saturday or Sunday. The Friday night is a separate shift, which is staffed by a doctor who has been working daytime Monday–Thursday. Monday–Thursday, the evening hours 16:00–19:00 are covered by a colleague working during the day, who, thus, prolongs the workday by 3 h. The doctors covering the on-call shifts are either specialists in surgery or residents from year two or later. A senior back-up consultant is available per telephone around the clock, and needs to be physically present within 30 min if needed. Every day, a junior resident is supporting the on-call doctor between 16:00 and 23:59, by covering all phone calls from the nurses of the Surgical Clinic’s inpatient wards. On-call work duties primarily imply medical responsibility for the inpatients of the Department of Surgery, while the emergency department is run by specialists and residents in emergency medicine, employed the emergency department.

During office hours, there are two operating rooms (ORs) designated for acute and semi-acute cases in general- and vascular surgery. From 16:00 until 21:00, one OR is available for acute general surgery, vascular surgery, and urology. From 21:00 until 7:30, one OR is distributed between acute general surgery, vascular surgery, gynecology/obstetrics, urology, orthopedics, and hand surgery.

### Data collection

At the Department of Surgery at Södersjukhuset, there is a local quality register including all patients who have undergone an appendectomy [15]. The patients are continuously identified through the local operation logistic software (*Orbit5*, TietoEvry, Kristianstad, Sweden), using codes from NOMESCO Classification of Surgical Procedures version 1.15: *JEA 00* (Appendectomy), *JEA01* (Laparoscopic appendectomy) and *JEA10* (Appendectomy with drainage) [16]. Procedure-related data are extracted into the register from *Orbit5* and other parameters are retrieved from electronic medical records (*TakeCare*, CompuGroup Medical, Helsinki, Finland).

Patients undergoing acute appendectomy between December 2015 and August 2022 were included in the current study. Method of surgical approach (laparoscopic, open, converted from laparoscopic to open in complicated cases) and eventual perforation was registered manually, using the operating surgeon´s medical chart entry. Postoperative data, including complications, readmission and death, was registered manually 30 days after surgery, using medical records from the hospital. Medical records from other hospitals and/or primary health care centers were also registered, as most healthcare providers in the region are using the same software for medical records (*TakeCare*). Surgical complications were defined as wound infection, intrabdominal abscess, paralytic ileus, mechanical small bowel obstruction, bleeding/hematoma, and others (e.g., urinary tract infection, wound dehiscence).

Data on hospital stay, such as length of stay, date, and time of arrival in the emergency department, were retrieved automatically from *TakeCare*.

Operation-related parameters were obtained automatically from *Orbit5*. Physical status was determined by the responsible anaesthesiologist, using *American Society of Anaesthesiologists* (ASA) assessment [17]. Time to surgery was defined as the elapsed time from the surgeon´s decision of appendectomy, until start of surgery. Length of surgery was defined as time from skin incision to skin closure.

Start of surgery was defined as the whole hour of the day when surgery was initiated (0–23). Start of surgery was further divided into 8-h blocks defined as *day* (8–15)*, evening* (16–23), and *night* (0–7). For example, a procedure initiated at 7:59 would be classfied as *night*, while surgery inititated at 8:00 would be classfied as *day*.

### Statistical analyses

For the statistical analyses, *IBM SPSS Statistics Version: 28.0.0.0(190)* was used. The exposures of interest were surgery at day, evening, and night, and the cohort was, thus, divided into three groups accordingly. Differences in patient characteristics of the three groups were compared using a two-tailed chi-square test for categorical variables and an ANOVA test for continuous variables. Categorical variables were presented as numbers and percentages, and continuous variables were presented as means and 95% confidence intervals (CIs).

Main outcome was postoperative complication registered within 30 days after surgery and secondary outcomes were readmission within 30 days, death within 30 days and length of hospital stay. Risk estimates of complication, readmission, and death were computed using uni- and multivariable logistic regression models with daytime surgery as reference, and presented as odds ratios (ORs) and 95% CIs. The multivariable model was adjusted for age, sex, surgical approach, ASA classification, perforation, time to surgery and length of surgery.

Timing of surgery and the effect on length of hospital stay were estimated in a multiple linear regression model, including the same covariates as in the logistic regression model, with daytime surgery as the reference.

A *P*-value of ≤ 0.05 was considered significant, for all statistical analyses.

### Ethical approval

Ethical approval of the study was obtained from the Swedish Ethical Review Authority (Dnr 2019-05976).

## Results

During the study period, 5120 patients were registered, of whom 59 were excluded due to being elective cases and 110 were excluded due to missing date of surgery. This left 4950 patients, of whom 2197 (44.4%) were women, eligible for analyses. In the analyzed cohort 97.2% underwent laparoscopic appendectomy, 1.5% were converted from laparoscopy to open appendectomy and 1.3% underwent open surgery without any attempt of prior laparoscopy. Mean procedure time for laparoscopy was 52.9 min and 108.3 min for cases who were either converted from laparoscopy, or open from the start.

Postoperative complications within 30 days occurred in 307 (6.2%) of the patients and was more common in men (7.0%) than in women (5.2%). The most common complication during the study was intraabdominal abscess (166 patients), followed by paralytic ileus (73 patients), wound infection (38), bleeding/hematoma (18), mechanical small bowel obstruction (9), and others (3).

**Fig. 1:**
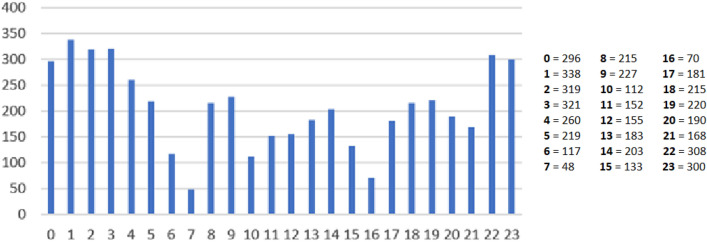
Number of appendectomies initiated by hour of the day

The distribution of initiated appendectomies was 1 380 (27.9%) in daytime, 1 652 (33.4%) in the evening and 1 918 (38.7%) in the night. Hour by hour, appendectomy was most commonly started between 01:00 and 01:59 with 338 cases (6.8%). The lowest number of initiated appendectomies was between 07:00 and 07:59 with 48 cases (1.0%). The hourly distribution of appendectomies is illustrated in Fig. 1.

The group of patients who underwent appendectomy during daytime had the highest mean age (33.34 years), the most men (57.4%) and had the highest fraction of patients with an ASA classification of III–IV (6.6%). The lowest mean age of patients was during the night (31.59 years), meanwhile most female patients (47.6%) and the lowest fraction of patients classified as ASA III–IV (4.1%) underwent evening surgery. Daytime surgery was associated with the longest mean time to surgery (8.59 h), mean length of surgery (60.46 min), and mean length of hospital stay (53.75 h). The shortest mean time to surgery was among patients operated in the evening (6.50 h), but the shortest- mean length of surgery (49.95 min) and mean length of hospital stay (46.33 h) was registered in patients who underwent surgery during the night. There were no significant differences in rates of only-laparoscopic surgery, perforation, complications, readmission, and death between the three groups. Detailed patient characteristics of the three groups are displayed in Table 1.

**Table 1:** Baseline characteristics for patients operated during day, evening, and night (continuous variables presented as means and 95% CI)

Variable	Day, *n* = 1380	Evening, *n* = 1652	Night, *n* = 1918	*P*
Age, years	33.34 (32.33–34.35)	32.54 (31.65–33.43)	31.59 (30.76–32.43)	0.028*
Male sex, *n* (%)	793 (57.4%)	866 (52.4%)	1094 (57.0%)	0.006*
Laparoscopy only, *n* (%)	1 338 (97.0%)	1 602 (97.0%)	1 873 (97.7%)	0.356
ASA III–IV, *n* (%)	91 (6.6%)	67 (4.1%)	84 (4.4%)	0.002*
Perforation, *n* (%)	340 (24.6%)	447 (27.1%)	543 (28.3%)	0.062
Time to surgery, h	8.59 (8.28–8.90)	6.50 (6.29–6.71)	7.09 (6.87–7.31)	< 0.001*
Length of surgery, min	60.46 (59.13–61.78)	54.63 (53.28–55.98)	49.95 (48.66–51.25)	0.006*
Length of stay, h	53.75 (51.08–56.42)	48.39 (45.91–50.86)	46.33 (44.11–48.54)	< 0.001*
Complication, *n* (%)	89 (6.4%)	111 (6.7%)	107 (5.6%)	0.335
Readmission, *n* (%)	52 (3.8%)	54 (3.3%)	58 (3.0%)	0.496
Death, *n* (%)	1 (0.1%)	2 (0.1%)	2 (0.1%)	0.914

*P-value of significance

Using uni- and multivariable logistic regression models, there were no significant differences in associated risk of postoperative complication or readmission within 30 days, between patients operated during day, evening or night. However, risk of readmission within 30 days was borderline significant (*P* = 0.068), with an OR of 0.70 for night-time surgery, in the multivariable model. There were, however, other covariates in the multivariable model, who were significantly associated with risk of complications and readmission. Male sex was associated with an increased risk of postoperative complications (OR 1.33) meanwhile an only-laparoscopic surgical approach was associated with a lower risk (OR 0.28). Perforation was associated with both complications (OR 6.95) and readmission (OR 4.24). Time to surgery and length of surgery were not associated with risk of complications, but had small yet significant risk estimates of readmission (OR 0.96 and 1.01 respectively). Results from the multivariable logistic regression are presented in Table 2.

**Table 2:** Multivariable logistic regression model: timing of appendectomy and associated risk of complications and readmission, presented as ORs and 95% CI

Variable	Complication	*P*	Readmission	*P*
Evening surgery^a^	0.99 (0.73–1.35)	0.960	0.77 (0.52–1.15)	0.206
Night surgery^a^	0.79 (0.58–1.08)	0.141	0.70 (0.47–1.03)	0.068
Age	1.00 (1.00–1.01)	0.556	0.99 (0.99–1.00)	0.167
Male sex	1.33 (1.03–1.71)	0.028*	1.11 (0.81–1.54)	0.515
Laparoscopy only	0.28 (0.18–0.43)	< 0.001*	0.71 (0.35–1.43)	0.333
ASA III–IV	1.50 (0.97–2.31)	0.068	1.52 (0.83–2.80)	0.174
Perforation	6.95 (5.31–9.09)	< 0.001*	4.24 (3.04–5.91)	< 0.001*
Time to surgery (h)	0.98 (0.95–1.00)	0.078	0.96 (0.93–1.00)	0.036*
Time of surgery (min)	1.00 (0.99–1.01)	0.64	1.01 (1.00–1.02)	0.032*

*P-value of significance

^a^Daytime surgery as reference

Death within 30 days was such a rare occasion within our cohort (0.1%, *n* = 5) and was evenly distributed around the clock (1 during day, 2 during evening, 2 during night). Using logistic regression models, there were no significant differences in associated risk of death between patients operated during day, evening or night, in either uni- or multivariable models. Also, the risk estimates were hard to interpret due to very large (yet insignificant) numbers for some covariates (ASA III–IV and perforation) in the multivariable model. Risk estimates for death are, therefore, not reported in any table.

Surgery during evening and night, as compared to daytime surgery, was associated with shorter hospital stay, using multiple linear regression. Evening surgery shortened hospital stay by 4.21 h; meanwhile, night-time surgery shortened hospital stay by 6.71 h. Aside from patient sex and length of surgery, all other independent variables included in the model had a significant effect on length of hospital stay. Results from the multiple linear regression are displayed in Table 3.

**Table 3:** Multiple linear regression model, timing of appendectomy and effect on length of hospital stay in hours

Independent variable	Coefficient	Standard error	*P*
Evening surgery^a^	– 4.21	1.60	0.008*
Night surgery^a^	– 6.71	1.54	< 0.001*
Age (years)	0.10	0.04	0.005*
Male sex	0.11	1.25	0.928
Laparoscopy only	– 69.05	3.83	< 0.001*
ASA III–IV	28.53	3.06	< 0.001*
Perforation	43.16	1.44	< 0.001*
Time to surgery (h)	0.65	0.12	< 0.001*
Length of surgery (min)	– 0.25	0.04	0.510

*P-value of significance

^a^Daytime surgery as reference

## Discussion

In our study of 4,950 acute appendectomies, there were no significant differences between the risk estimates of postoperative complications, readmission or death, regardless of when surgery was performed. However, surgery during evening or night was associated with shorter hospital stay.

Two similarly designed studies with similar objectives had findings that correspond to ours. Neither Mönttinen et al. [13] nor Shah et al. [14] found any differences between the risk estimates of complications after acute appendectomies performed during different times of the day. Rates of infectious complications, bleeding, perforation and conversion from laparoscopic to open surgery were similar to those reported by Mönttinen et al. [13]. Meanwhile, rates of deeper infection (3.35% vs 0.80%), conversion from laparoscopy (1.50% vs 0.20%), perforation (26.87% vs 8.49%), and readmission (3.31% vs 1.00%) were considerably higher in our setting than compared to Shah et al. [14]. This could plausibly be explained by a considerably lower amount of previously healthy patients in our setting (62.9% classified as ASA I vs 93.5% without comorbidities), meanwhile mean age was similar (32.39 years vs 32.23 years). However, our study comprised patients of ≥ 10 years, meanwhile the study by Shah et al. was made up by adults of ≥ 18 years, upon which age comparison between the two should be done cautiously.

When assessing the hourly distribution of appendectomies during the day, two obvious low-points are identified at 7:00 and 16:00. This is most likely described by the transition from on-call hours to office hours and vice versa. It is probably rare that someone starts a non-mandatory procedure if it is unlikely that they can finish before their shift ends. A similar down-going trend is noticed at 21:00, which is the time when the evening-working or and anaesthesiology nurses end their shifts, and the night-working staff start theirs. Regarding the decline at 10:00, we have no obvious explanation, but it could be hypothesized that other expectedly difficult surgical cases are not initiated during the later hours of the night, but instead postponed until early daytime when the highest possible collective competence is present. While such cases are taken care of early in the morning, it is likely that expected easier cases of acute appendicitis are postponed until on-call hours. Patients with other acute surgical conditions are likely to be prioritized over patients with acute appendicitis during daytime, unless priority of the appendectomy for any reason is high. Since laparoscopic appendectomy in general is an uncomplicated procedure that all on-call working doctors master, it should be safe to postpone until evening or night. The surgeon´s fatigue during night does not seem to have a negative impact on outcome in our setting. Even though other studies have similar findings [13, 14], it should be noted that our unit work by what internationally seems to be a relatively favorable schedule during night shift weeks, with work every other night and with off days between the nights, which should enable adequate recovery.

When treating any urgent inpatient, regardless of disease, the main objective is first and foremost the clinical outcome. In our study, this was not affected by when acute appendectomy was performed. However, a secondary objective in urgent inpatients should be to shorten hospital stay since this is beneficial for the patient in question, other patients in need of a physical inpatient bed, and also in an economical sense for the hospital. In our setting, hospital stay was shortened by roughly 4.2 h or 6.7 h, when acute appendectomy was performed during evening and night, respectively. Since the annual volume of acute appendectomies during the study period was roughly 730, appendectomies performed after day-hours could reduce the yearly number of inpatient days by around 125 – 205, in our setting. This should, aside from surgical outcome, be an incentive to perform acute appendectomies around the clock. Mönttinen et al. [13] carried out their study in a similar setting in a neighboring Scandinavian country, and had findings with patterns of hospital stay similar to those in in our study, with shorter stay when surgery was carried out at night. Meanwhile, Shah et al. found opposite relationships, with the shortest mean hospital stay for appendectomies performed between 7.00 am and 1.00 pm (1.8 days) and the longest mean hospital stay for those performed between 7.00 pm and 1.00 am (2.6 days). However, the latter study was carried out in a Mid-Eastern country and one could hypothesize that Swedish working routines have more in common with Finnish ones, than with Qatari. This could possibly explain this difference.

In the current study, there were several non-timing-related factors associated with the risk of postoperative complications. A surgical approach with laparoscopy only was associated with a lower risk of postoperative complications, meanwhile perforation and male sex was associated with higher risk estimates. As almost all acute appendectomies are initiated laparoscopically at our center, the need of open surgery in a patient would indicate a particularly complicated procedure, which in itself should explain why the risk of complications would be higher. The need of conversion from laparoscopy to open surgery has previously been found to associate with an increased risk of complications postoperatively [18]. The finding of perforated appendicitis and the increased risk of postoperative complications also goes in line with previous literature, as perforated appendicitis is more commonly associated with postoperative morbidity [18, 19]. The rate of perforated appendicitis in our setting (26.9%) was in the range of what has been reported in previous studies [13, 20, 21]. Male sex has previously been described to associate with increased risk of appendix perforation [18, 22, 23] and it has also been reported that boys are at higher risk of postoperative infectious complications than girls [24].

A somewhat unexpected result was that every hourly delay of time to surgery was associated with a 4% decreased risk of readmission in the multivariable logistic regression model. This could possibly be mediated by the fact that patients who were given high surgical priority would receive surgical treatment earlier and could also be more likely to demand for readmission later, due to a more severe disease than among patients who received lower surgical priority. The model was admittedly adjusted for perforation of the appendix, which was more common in patients who had surgery within 6 h than in patients with surgery after 6 h (30,7% vs 23,0%), but not for other factors that also could indicate a severe degree of inflammation, such as affected vital signs, general peritonitis or gangrenous appendicitis, as these parameters were not available within the register.

A factor to consider when planning for acute appendectomy aside from the safety of the procedure and the length of hospitalization is the patient´s discomfort while waiting for surgery. The longer the wait, the longer time in pain, and, thus, probably a relatively increased level of preoperative analgesic treatment, partially opioids. Since opioid treatment comes with several side effects [25], it should be desirable to reduce the patient´s consumption whenever possible. Avoiding unnecessary delay by operating around the clock should be effective to obtain this objective. However, we have not been able to assess the levels of opioids used pre- and postoperatively, in the current study setting.

### Strengths and limitations

A strength of the current study is the size of the cohort, including close to 5 000 patients. The annual volume of acute appendectomies was roughly 730 patients, meaning 2 cases per day. Another strength within our setting is the data source that is the local appendectomy register, with a 100% inclusion rate of all appendectomies carried out at the clinic. A limitation within the retrospective register-based cohort design is the inability to assess causal relationships.

The lack of specified background information on each patient, e.g., BMI, smoking status and other diseases, was another limitation in the current study. However, ASA classification could be used as a surrogate measure to determine physical status, although not specifically. Also, values of inflammatory blood markers such as CRP and leukocytes were not routinely extracted from the patient journals in the appendectomy register. We did not find it mandatory to collect these data subsequently, since it is likely that the surgeon has taken inflammatory markers at least partially into account when determining the level of urgency for the procedure. Therefore, time to surgery could act at least as a blunt surrogate measure for preoperative assessment of inflammatory degree.

In our study, perforation of the appendix was an independent risk factor associated with postoperative complications. Perforated appendicitis is routinely treated with postoperative antibiotics for five days in our clinic, initially intravenously (either Piperacillin–tazobactam or Cefotaxime + Metronidazole), followed by oral treatment (generally Ciprofloxacin + Metronidazole). However, compliance to this treatment has not been logged in the local register, and has not been registered subsequently either. This uncertainty constitutes for potential bias, as patients with perforated appendicitis could possibly have been undertreated, thus contributing to higher rates of complications.

## Conclusion

In our setting, acute appendectomy was equally safe in terms of postoperative clinical outcome, independent of what time during the day surgery was performed. However, surgery during evening and night was associated with significantly shorter hospital stay as compared to daytime surgery. Thus, if logistically possible, acute appendectomy should be performed around the clock, without unnecessary delay, to reduce the time of symptoms as well as the time of hospitalization. However, a major part of acute appendectomies globally are carried out in countries where financial resources are scarce and do not support a surgical team as well staffed around the clock, as described in the current study. Our results suggest that even if non-delayed acute appendectomy is desirable, postponement of the procedure from night until daytime should be safe in terms of surgical outcome, and therefore a feasible option. Future studies should look into how opioid consumption levels, as well as hospital expenses, are affected by acute appendectomies carried out at different times of the day.

## Data Availability

Due to ethical restrictions, supporting data are not available for third party.
